# Potential of Peptide Nucleic Acids in Future Therapeutic Applications

**DOI:** 10.15171/apb.2018.064

**Published:** 2018-11-29

**Authors:** Soheila Montazersaheb, Mohammad Saeid Hejazi, Hojjatollah Nozad Charoudeh

**Affiliations:** ^1^Stem Cell Research Center, Tabriz University of Medical Sciences, Tabriz, Iran.; ^2^Molecular Medicine Research Center, Tabriz University of Medical Sciences, Tabriz, Iran.; ^3^Department of Pharmaceutical Biotechnology, Faculty of Pharmacy, Tabriz University of Medical Sciences, Tabriz, Iran.; ^4^Anatomical Sciences Department, Faculty of Medicine, Tabriz University of Medical Sciences, Tabriz, Iran.

**Keywords:** Antigene, Antisense, Cancer, Gene Therapy, Peptide Nucleic Acid, Splicing

## Abstract

Peptide nucleic acids (PNA) are synthetic analog of DNA with a repeating N-(2-aminoethyl)-glycine peptide backbone connected to purine and pyrimidine nucleobases via a linker. Considering the unique properties of PNA, including resistance to enzymatic digestion, higher biostability combined with great hybridization affinity toward DNA and RNA, it has attracted great attention toward PNA- based technology as a promising approach for gene alteration. However, an important challenge in utilizing PNA is poor intracellular uptake. Therefore, some strategies have been developed to enhance the delivery of PNA in order to reach cognate site. Although PNAs primarily demonstrated to act as an antisense and antigene agents for inhibition of transcription and translation of target genes, more therapeutic applications such as splicing modulation and gene editing are also used to produce specific genome modifications. Hence, several approaches based on PNAs technology have been designed for these purposes.

This review briefly presents the properties and characteristics of PNA as well as different gene modulation mechanisms. Thereafter, current status of successful therapeutic applications of PNA as gene therapeutic intervention in different research areas with special interest in medical application in particular, anti-cancer therapy are discussed. Then it focuses on possible use of PNA as anti-mir agent and PNA-based strategies against clinically important bacteria.

## Introduction

**Figure 1:**
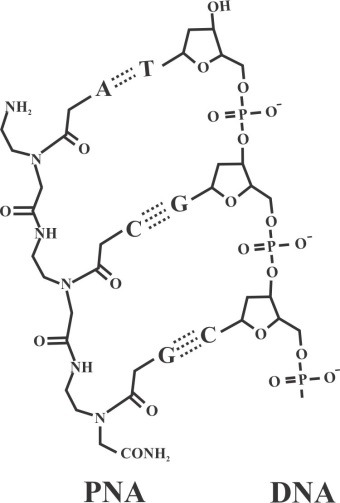
Comparison of chemical structure of PNA and DNA backbones.^2^

**Figure 2:**
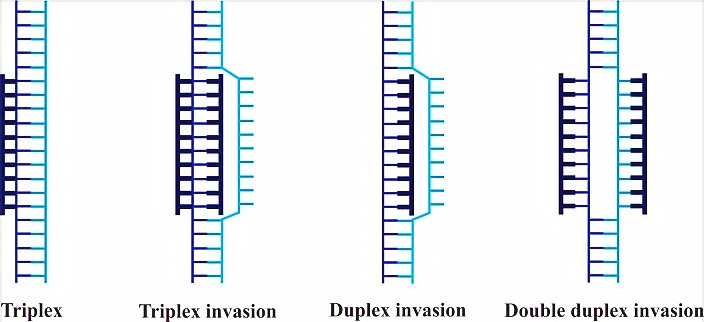
Schematic drawing of PNA binging modes, targeting to dsDNA. DNA is drawn as a ladder and PNA molecules are in thick structures. Triplex complex is formed by cytosine rich PNA targeting to homopurine target. Triplex invasion structure leads to the formation of a loop. In this structure, one strand of PNA binds based on Watson-Crick rule, and another strand binds through Hoogsteen bond. Duplex invasion has no sequence constraints. Double duplex invasion is formed by PNAs containing modified bases.^3^

Peptide nucleic acid (PNA) is a non-charged DNA-like molecule that is remarkably similar to DNA and RNA both in intramolecular spacing and geometry (Figure 1). Unlike DNA or other DNA analogues, the entire sugar-phosphodiester is replaced by a neutral charge and achiral pseudopeptide backbone to which the bases are attached via a methyl carbonyl linker.^1^ One consequence of this neutrality is the lower intrinsic electrostatic repulsion against complementary oligonucleotides, resulting in greater hybridization strengths. PNA has the capability to hybridize with high affinity and sequence specificity to complementary DNA or RNA sequence, obeying the Watson-Crick hydrogen-bonding scheme in which adenine pairs with thymine via two hydrogen bonds and cytosine forms three hydrogen bonds with guanine. PNA molecules with mixed sequences form duplexes with complementary DNA and RNA based on Watson–Crick rule. In addition, PNA can form conventional triplex structure through Hoogsteen binding in the major groove of helix of the double stranded DNA. Homo-pyrimidine PNA can form triplex invasion structure with DNA when targeting to homopurine sequence in duplex DNA (Figure 2). In this structure, one PNA strand hybridizes to its complementary DNA through Watson-Crick hydrogen bonds via antiparallel orientation, while the second PNA strand binds to the same sequence through Hoogsteen hydrogen bonds in parallel form.^2^ Because of sufficient inherently flexible polyamide structure, PNA oligomers display higher affinity and specificity when targeting DNA and RNA strands (or PNA itself) compared to natural DNA or RNA.^3^

Since the introduction of PNA by Nielsen, Buchardt, Egholm and Berg (1991),^4^ it has caught attention in many fields, including molecular biology, biotechnology, biosensor systems,^5^ drug discovery, genetic diagnosis and development of gene therapeutic agents.^6^ In addition, PNA can be used as a diagnostic tool for detection of oncogenes through distinction between the wild type and the mutant target.^7^ Owing to the peptide-like chemical structure, the synthesis of PNA is similar to peptide protocols. In this way, PNA molecules are easily synthesized by well-established solid-phase manual or automated methods using either tBoc (tert-butyloxycarbonyl group) or Fmoc (9-fluorenylmethoxycarbonyl group) solid phase and purification with high-performance liquid chromatography (HPLC).^8^

### Attractive properties of PNA 

PNA possesses several peculiar and unique physicochemical properties as compared to the natural counterparts. The unnatural backbone of PNAs renders charge neutrality, eliminating electrostatic repulsion which has a negative effect on binding affinity, and causes excellent hybridization affinity to target sequence. An additional outcome is that PNAs bind independently of medium salt concentration, whereas low ionic strength reduces the stability of DNA–DNA duplexes. Also, PNAs show stability at acidic pH (pH 4.5~6.5), while DNA is prone to depurinate at lower pH. Detailed results have revealed that PNAs have higher thermal melting temperature (Tm) compared to corresponding DNA–DNA or DNA–RNA duplexes as Tm of a PNA/DNA duplex for per base pair is 1° C higher than DNA–DNA duplex.

As stated above, one crucial consequence of unnatural backbone is resistance to nuclease and protease-mediated degradation in serum and cell extracts, extending the life time of this oligomer both *in vitro* and *in vivo*.^2^ Noticeably, PNAs display very good mismatch sequence discrimination,^9,10^ and strong binding affinity towards DNA and RNA, making this oligonucleotide mimic an interesting third generation oligonucleotide analogue with potential therapeutic applications.^11^ PNA is capable of blocking the activity of DNA and RNA polymerases, telomerase, reverse transcriptase, endonucleases and transcription factors. Commensurate with these statements, the DNA/RNA recognition capacity has made PNA indeed a promising candidate for therapeutic application through modulation of gene expression via antigene and antisense mechanism (Table 1).^12,13^

### Limitations of PNA Technology

Despite the remarkable properties of PNA along with simplicity of synthesis and high chemical and biological stability which make PNA as a potential candidate to develop effective gene therapeutic agents, there are basic challenges still remaining for clinical application. Poor water solubility of this oligomer due to neutral character makes it to have a tendency to form globular structures. To improve the low water solubility of PNA oligomer, some modifications should be applied including addition of positively charged residues (at the terminals) and conjugation to negative charge molecules such as DNA.

Another significant challenge of this hydrophilic oligomer is the poor cellular uptake. To combat limitations in crossing of PNAs and achieving sufficient intracellular delivery, several modifications can be performed to enhance cellular delivery. Without passing cell membrane, PNAs cannot interact with target sequence and therefore have no biological activity.^14^

### PNA delivery approaches

To make a successful PNA-based technology, there is a need to find effective methods to enhance the access of PNAs into intracellular space.^15^ A variety of methods such as spontaneous uptake in some type of nerve cells, electroporation, membrane permeabilization with streptolysin O, cationic lipids, and attachment of PNAs to cationic cell-penetrating peptides (CPPs) have been used for delivery of PNAs into the target cells.^16^ The latter approach is an extremely efficient transport carrier for moving across cell membranes. It has been shown that CPP has no significant additional cellular toxicity especially for therapeutic application. Most known examples of CPPs are positively charged amino acids including arginine and lysine which interact with glycoproteins having negative charge on the cell surface. Of note, due to guanidine moiety in arginine residue which can interact with negative groups, this amino acid is more effective than lysine. Although the mechanism of CPP-mediated delivery is still not very clear, CPPs can release their cargo into cells using two distinct mechanisms including endocytosis and direct translocation. Since PNA needs to reach the target site for modulating gene expression, the internalized cargo must be released from endosomal compartments into the cytoplasm. It is noteworthy that co-treatment with endosome disruptive agents such as chloroquine can enhance the release of entrapment cargo, and thus dramatically increase the effects of PNA-conjugates.^17^

*In vivo* delivery of PNA still has many limitations. As the target site of PNA molecule is located within the cells, so PNA must enter into the cytoplasm or nucleus with assuring circulation time and reasonable excretion until reach to the target site. As noted, PNAs are hydrophilic compound and do not cross cell membranes, therefore are excreted very rapidly with half-life of approximately less than one hour. As a result, to achieve sufficient bioavailability of PNA molecules, it must be chemically modified to assure *In vivo* delivery efficacy. Detailed reports revealed that conjugation to CPP peptides can improve *in vivo* efficacy. For instance, it has been shown that PNA conjugated to oligoarginine has moderate activity in muscles in the mdx mice. Notably, nanoparticle PNA-based strategies can be used for efficient delivery of PNA to target sites.^18,19^

### Advantage and disadvantage of PNA in gene-based therapy

Gene therapy uses foreign genes or short oligonucleotide sequences as therapeutic agents. Several approaches can be used for correcting of defect genes such as addition of functional alleles and correction via homologous recombination, introduction of suicide genes and prodrugs and inhibition of gene expression through synthetic oligonucleotides. There is particular interest in using oligonucleotides due to specificity to cognate site and non viral-based strategies. In conventional gene therapy, viral vectors are often used for gene delivery that may cause some risks including, stimulation of immune reaction and more importantly insertion of oncogene.^20^

PNAs are more efficient in terms of gene modulation, compared to traditional oligonucleotides such as phosphorothioate. For instance, one study showed that higher concentration of PNA is required for inhibition of gene expression than propyne-phosphorothioate oligonucleotides. Even supposing that higher concentrations of PNA is necessary to obtain reasonable antisense effects, the unique properties of PNA such as resistance to enzymatic digestion, higher stability, and specificity of PNA molecule to target sequence make it a powerful choice for gene therapy.^1^

## Gene modulation mechanisms of PNA molecules

### Antigene applications of PNA

**Figure 3:**
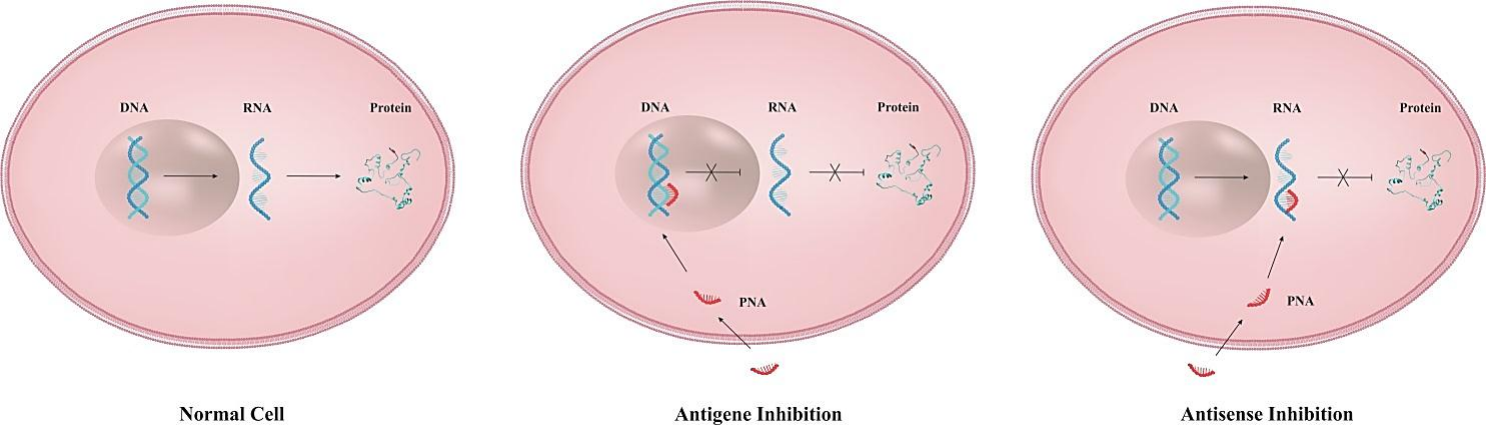
Schematic illustration of PNA-based strategy as antigene and antisense agents. Antigene PNA binds to complementary sequence in DNA and inhibits transcriptional process while antisense PNA hybridize to target sequence in mRNA and prevent translation procedure.

PNAs have the ability to hybridize to complementary sequences at the DNA and interfering with the transcription of the target gene and as a consequence, reducing the mRNA level (Figure 3). In fact, PNA is capable of arresting transcriptional processes by virtue of its ability to form different complexes with dsDNA such as a stable triplex structure, strand invasion or strand displacement complexes leading to structural hindrance of RNA polymerase, as depicted in Figure 2.^21^ Triplex-forming PNA can hamper the initiation of transcription by preventing duplex DNA unwinding or inhibition of transcription factor binding to the promoter region of target genes. Moreover, triplex-invasion structures within the transcribed region on the template strand can block the elongation of RNA polymerase along with the appearance of truncated RNA transcripts, inducing transcriptional inhibition.^22^ Targeting chromosomal DNA is less effective due to difficult access to target sequence under physiological conditions. For instance, the presence of cations has potential to stabilize the DNA double strands, resulting in the reduction of helix invasion by the PNA. On the other hand, specific gene modulation via antigene mechanism is advantageous over antisense approaches, in that one (or two) copy of target sequence is evoked compared to many targets of mRNAs present in the cell.^23^

### Antisense applications of PNA

**Figure 4:**
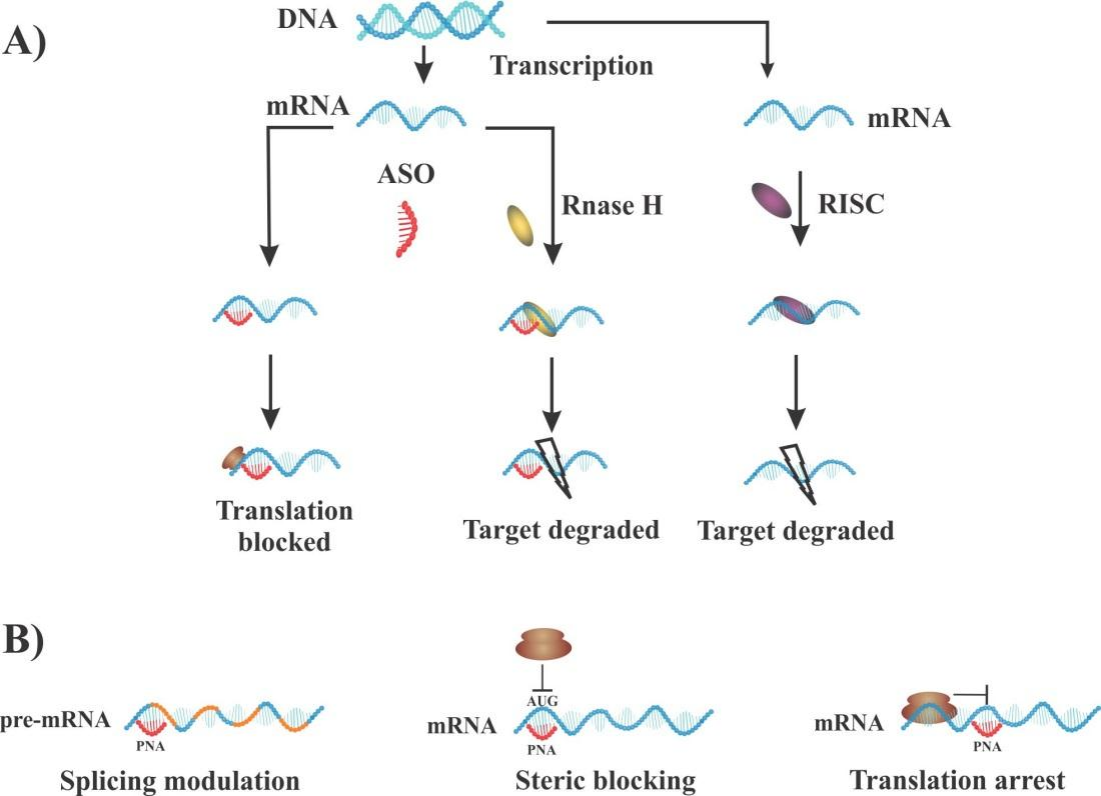
Some mechanisms involved in modulation of gene suppression: A) Antisense activity and RNA interference mechanisms. B) Inhibitory effect of antisense PNA via different approaches. PNAs have the ability to target AUG regions (translational start codon) or 5´UTR, where the starting point of ribosomal assembly occurs and block process, whereas triplex forming PNAs can inhibit elongation process via targeting to coding region. ASO: antisense oligonucleotide.

In the case of RNA interference, PNA molecules mediate inhibitory effects on translational process via steric blocking mechanisms. Since PNA oligomers neither activate ribonuclease H (RNase H) nor enter RNA-induced silencing complexes (RISC) (Figure 4A), thus, antisense role of PNA may rely on mechanistic interference via different mechanisms rather than degradation of mRNA. PNAs hamper translation process through RNA transport to cytoplasm, elongation process, and translation initiation such as ribosome assembly.^24^ Of note, inhibitory effects depend on hybridization strength and good accessible sequence on the mRNA molecule. Interestingly, it has been observed that duplex-forming PNAs with mixed sequence have the highest potency when targeted to AUG regions (translational start codon) or 5´terminus of mRNA (5´UTR), where ribosomal assembly occurs. Alternatively, homopyrimidine triplex-forming PNAs inhibit elongation process, leading to truncated products, whereas as noted above, duplex PNA can efficiently prevent the ribosomal assembly rather than arresting elongation (Figure 4B). Detailed data demonstrated that complete blockage of translation can be efficiently achieved by applying two or more duplex-forming PNAs. Overall, a crucial rule is considering the 5´UTR and AUG as suitable regions to obtain good antisense activity for duplex forming PNA.^25,26^

Certainly, ribosomes of eukaryotic cells recognize the translation initiation codon through scanning the mRNA sequence from the 5´-cap site to AUG codon. During the scanning process and before recognition of start codon, the assembly of ribosome has not completely occurred and is most likely not connected with the mRNA. As a result, the translation machinery is a good target to block due to accessibility of PNAs to cognate regions.

### Modulation of splicing with PNA-based strategy 

**Figure 5:**
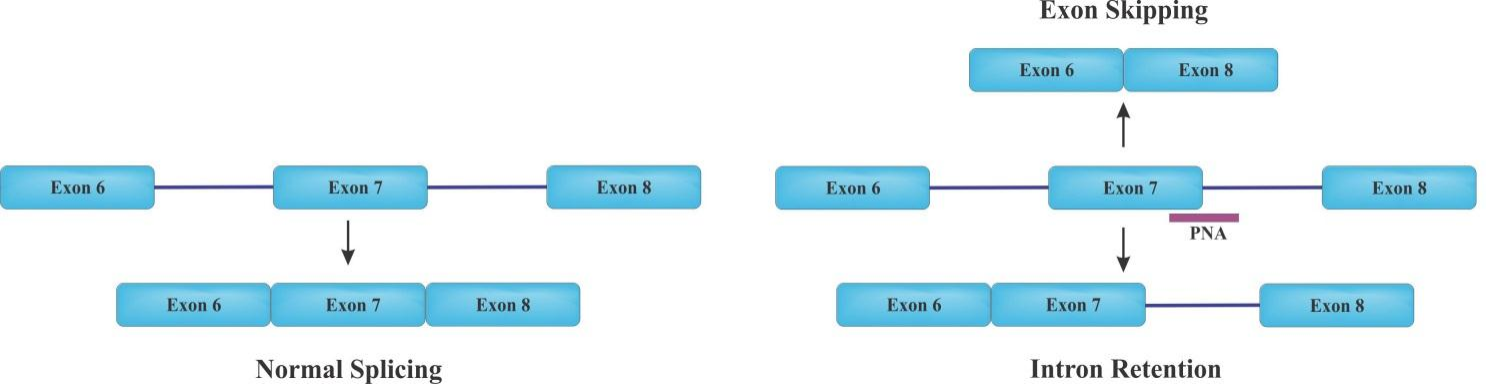
Splicing pattern of pre mRNA. Left: Normal splicing is shown by joining of exons to generate a spliced mRNA transcript. Right: Modification of splicing by antisense PNAs leading to exon skipping or intron retention. Blue rectangles, exons; lines, introns.

Interfering the splicing pattern is another important application of antisense PNAs, targeting and blocking specific intron-exon splice junctions within the pre-mRNA molecule. Upon targeting a PNA molecule to intron–exon junction sites, splicing machinery (spliceosome) either skips one exon (or more) or may retains intron sequence. As depicted in Figure 5, intron retention leads to the production of nonfunctional mRNA containing an intron, which is susceptible to destabilization. On the other hand, exon skipping produces an alternative protein similar to alternative splicing procedure. Since several diseases result from shifting in the splicing patterns of pre-mRNA, so interference with splicing procedure may create novel possibilities for drug development.^27^ The process of pre-mRNA splicing is carried out by a very large ribonucleoprotein (RNP) complex through the removal of the intronic sequences accompanied by joining the exons at intron-exon junctions. This process is mediated by directing the spliceosome to correct splice sites at the intron-exon junctions including the 5´ and 3´ junction sites and the branch point. Employment of PNA-based technology can block correct splicing sites, which is critical for splicing mechanism.^28^

Alternative splicing can produce multiple mRNA isoforms from one gene and thus, leads to the generation of proteins with different functions. Many studies demonstrated that alternative splicing can be used to alter gene production based on physiological condition. For instance; human genes containing introns can undergo alternative splicing to adjust the desired output.^29^ In addition, numerous therapeutic approaches are available to correct aberrant splicing in many disorders. Altering the splicing pattern of pre-mRNA can be done by antisense oligonucleotides in various disorder-related genes such as tau, c-myc, IL-5R, bcl-x, dystrophin, and ß-globin for obtaining therapeutically favorable results.^30,31^ Overall, PNA targeting pre-mRNA sequences can efficiently modulate splicing process to obtain desirable outcome.^32,33^

### PNA-based Gene Editing Approach

**Figure 6:**
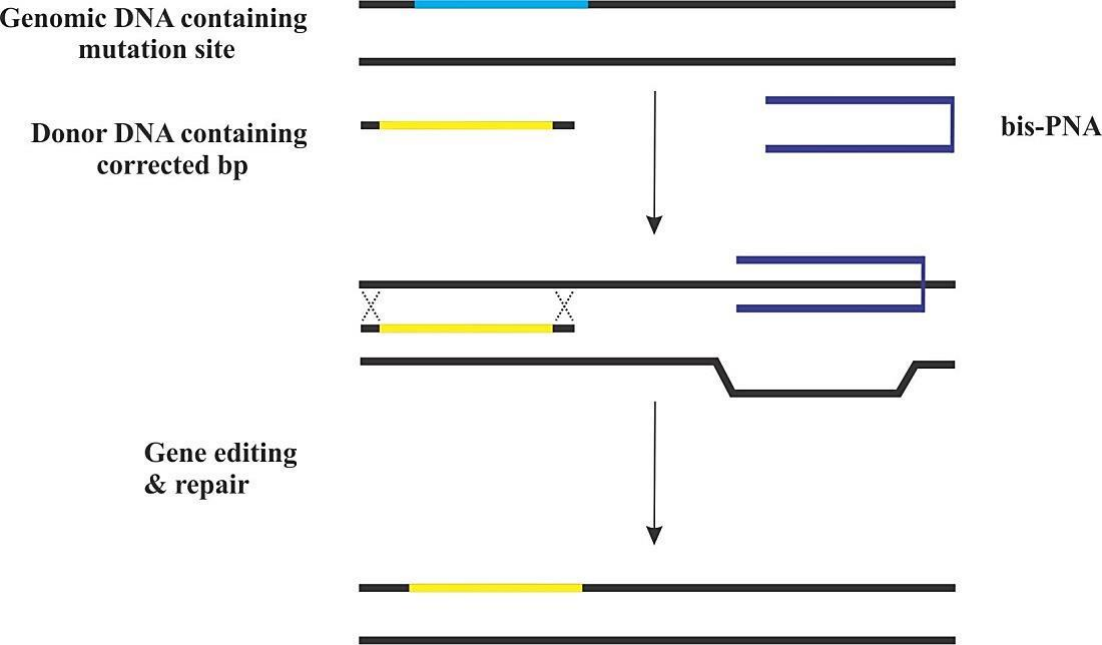
Schematic illustration of PNA-mediated gene editing. Triplex forming PNA can distort helical structure of DNA, inducing endogenous DNA repair in presence of DNA donor containing corrected nucleobases. The ability of PNAs in gene editing and repair comes directly from binding to cognate sites, provoking endogenous editing system.^81^

Triplex-forming PNAs are effective tools to induce homologous recombination in the presence of donor DNA. For achieving this process, two PNA molecules are attached together by a linker, forming a bis-PNA. One strand of bis-PNA binds in the anti-parallel orientation based on Watson-Crick rule, and the other strand is attached via Hoogsteen bond in the parallel direction to dsDNA, making triplex formation. This structure leads to distortion of DNA helical, which has the ability to provoke DNA repair system, and results in the recombination of “donor” DNA into the desired target near binding site of PNA (Figure 6). This technology for *in vivo* site-specific gene modification is a novel approach to treat human disease. Other gene editing technologies are based on exogenous nucleases activity such as CRISPR/Cas, they may possibly create breaks in dsDNA, whilst PNA-mediated gene editing relies on repair pathways with high fidelity, thus reducing the error of end-joining which can cause additional mutations.^14^

## PNA as a potential therapeutic agent for cancer therapy

Cancer is a major health problem in the world and is recognized mainly as genetic disease resulting from mutations of oncogene and tumor suppressor genes. The goal of cancer therapy is to find and develop novel strategies for modulating the expression of cancer-related genes using selective anticancer agents with no cytotoxic effects as observed in conventional chemotherapy.^34^ It has been shown that antigene and antisense strategies can be used to modulate transcription and translation of tumor genes. Moreover, numerous *in vitro* and *in vivo* experiments have already demonstrated the ability of PNA in controlling the dynamics of tumor gene expression. In accordance with a plethora of experiments, telomerase is found overexpressed in approximately 90% of human cancers, thereby being responsible for the immortalized cell phenotype. Of note, the modulation of this enzyme can be touted as an attractive target for cancer therapy. Besides, PNA *per se* has potential to hybridize to the RNA template region of telomerase and inhibit the catalytic activity of the enzyme.^35^ Overall, these results point out PNAs may be valuable tools for target-directed anticancer agents.

Bcl-2 is a 26-kD protein, which has a key role in the dynamic of apoptosis. Overexpression of Bcl-2 correlates with prolonged survival of cells, tumor development, poor prognosis and resistance to anticancer therapies. With this background, the control of bcl-2 expression is an interesting approach in cancer therapy. They examined various PNAs targeted to different regions of mRNA sequences. *In vitro* investigations further showed that the synthesis of bcl-2 protein was blocked through PNA targeted to 5´UTR and the AUG codon of human B-cell lymphoma in a dose-dependent pattern. Moreover, the combined use of both PNAs produced complete inhibition, whereas partial translation inhibition was seen with single PNA usage. Also, a triplex forming bis-PNA was capable of binding to the template DNA strand of the bcl-2 gene and specifically suppressed the transcription of the gene with the presence of truncated product. Overall, PNA-based approach was able to selectively manipulate both translation and transcription of bcl-2 gene *in vitro*.^36^

Chronic myeloid leukemia (CML) is a malignancy, resulted from translocation of chromosomes 9q and 22q, the Philadelphia chromosome (Ph), and generate the bcr/abl oncogene. This gene produces a protein (p210 ^BCR/ABL^) with high tyrosine kinase activity, leading to cell proliferation and malignant behavior. Thus, the unique sequence of *bcr/abl* is an attractive target for antisense therapy in CML disease. The incubation of CML cells with13 mer antisense PNA targeted to b2a2 junction in mRNA contributed to 35% decline in the level of p210^BCR/ABL^protein compared to control group. Also, anti-b2a2 PNAs have a strong antiproliferative effect in a dose dependent manner from 26% to 50% at 5 and 10 µM, respectively, displaying cell growth arrest.^37^

Acute promyelocytic leukemia (APL) is characterized by expression of the promyelocytic leukemiahetinoic acid receptor alpha gene (PML/RARa;PR). Fusion protein PML/RARα exists in all cases of APL and is generated by translocation of t (15; 17) chromosome. In this cancer model, the possibility of translation inhibition of the fused oncogene was tested by three different antisense PNAs targeting to first AUG start codon and second AUG and 5´UTR region of the fused oncogene sequence. Data showed PNAs directed against 5´UTR had the highest inhibitory activity compared to other ones. In fact, the limited effect of blocking was observed when PNAs used separately and a complete inhibition was achieved by the combination of these PNAs.^38,39^

Myc proteins play a key role in cancer. Overexpression of this protein is often correlated with DNA hypermethylation, higher cell growth rate, genomic instability and immortalization which lead to aggressive behavior of tumors.^40^ Commensurate with these statements, MYCN gene can be considered as an important target for cancer therapy with novel therapeutic inhibitors such as PNA to manage proliferation and apoptosis pathways and reduce the malignant behavior of the tumor.^39,41^ The possibility of PNA-based strategy for inhibition of MYCN transcription was previously examined by Tonelli and colleagues. They used an antigene PNA linked with nuclear localization signal peptide (NLS) targeted against a unique sequence of MYCN DNA (antisense DNA strand of exon 2) in six different cell lines of human neuroblastoma. The *in vitro* data showed a significant inhibition of MYCN expression by the antigene PNA in MYCN-amplified and MYCN-unamplified/low-expressed cell lines, leading to a reduction in cell proliferation rate, although no decrease was seen in MYCN-unamplified/unexpressed cell lines Compared with an antisense PNA, the inhibitory effect of the antigene PNA on MYCN gene expression showed stronger and longer inhibitory effect.^42^

Burkitt’s Lymphoma (BL) cell is characterized by upregulation of the c-myc oncogene due to translocation of chromosome 8 to chromosome 14, proximal to Eµ enhancer within the Ig heavy chain locus that can induce transformed features. Therfore downregulation of translocated c-myc with a specific PNA, can reduce transformed properties in BL. Given that translocated c-myc is under Eµ enhancer control, PNAEµ targeting to enhancer sequence seem to be a potential therapeutic agent*.* In a preliminary experiment, evaluation of pharmacokinetic parameters for PNA to an in vivo model system in severe combined immunodeficiency mice (SCID) demonstrated that after administration of PNA, it can persist intact in an *in vivo* system for prolonged time about 600 minutes. In this regard, PNA targeted to the Eµ enhancer region located on chromosome 14 caused selective downregulation of only translocated c-myc not the normal allele.^43^ Furthermore, using mice inoculated with the BL system demonstrated the potential capacity of PNAs to specifically and selectively block BL cells expansion and downregulation of c-myc expression.^44^

In addition, in a study, Cutrona and co-workers showed the effect of an anti-gene anti-myc PNA conjugated to a NLS in (BL) cell line. The authors used a 17-mer anti-myc PNA targeted to a unique sequence at the beginning of the second exon of the gene. The results indicated that PNA with 10 mM concentration achieved maximum inhibition of MYC expression (75%) and approximately (25%) reduction in cell viability, demonstrating that PNA specifically down-regulates the transcription of c-myc via binding to the double-stranded DNA *in vitro*. In consequence, PNAs are capable of inhibiting transcription of the targeted gene *in vitro* as well as *in vivo*, offering new specific therapeutic approaches to cure neoplasia.^45^

## PNA as a splicing modulator in clinically relevant approaches

The erbB2 gene is amplified in approximately 20–35% of human breast cancers and other cancer types. Several studies indicated that overexpression of this gene is accompanied by the higher aggressiveness of the tumor and more importantly development of resistance to anti-estrogen treatment and chemotherapeutic strategies.^46^ It has been shown that the application of antisense technology may be a useful approach towards treatment of breast cancers by targeting the translation start codon or the 3'-UTR region of the Her-2 mRNA to suppress the expression of Her-2 oncoprotein.^47^ In line with this hypothesis, Nielsen et al. examined several antisense PNA oligomers targeted to intron-exon junction sites to interfere with correct splicing of erbB-2 pre-mRNA in two cancer cell lines. Since exon 19 codes ATP catalytic domain of Her-2, PNA oligomers are targeted to the 5' exon-intron junction site to induce skipping of exon 19 which harbors many of the Her-2 cancer-related mutations. Considering the flexibility of the splicing machinery,^48^ obstruction of the 5' site could shift the spliceosome to another available splice site such as the 5' splice site of exon 20. Although the mechanism of skipping by PNA is not clear, it can be due to steric hindrance of pre-mRNA along with misleading of spliceosome through obstructing the correct splice site. The result demonstrated splicing redirection of Her-2 pre-mRNA by full-matched antisense PNA via skipping of erbB-2 exon 19. PNA-mediated activity can potentially be developed as anticancer strategies.^49^

The mdm2 oncogene encodes a nuclear phosphoprotein that inactivates the function of the p53 tumor suppressor protein. This oncogene is overexpressed in a wide variety of cancers.^50^ In cancer therapy, mdm2 down-regulation is a promising strategy which might lead to the induction of p53. In agreement with this claim, Nielsen et al. obtained inhibition of mdm2 mRNA translation by PNA targeting to initiation site (AUG), whereby p53 levels increased.^51^ Therefore, the mdm2 oncogene is an interesting antisense target for developing novel therapeutic agents and anticancer drugs. As mentioned above, PNA molecules were found to have a potent capability to target intron-exon junction sites. Again, Nielsen *et al*. designed several PNA molecules to down-regulate full-length mRNA of mdm2 or alter splice variants in human JAR cells through modulating splicing patterns. PNA oligomers tested toward the 5' or 3' splice sites in intron2 of mdm2 pre-mRNA inhibit splicing by generating a larger mRNA having retained intron 2. Among the PNAs, the most efficient is a complement to 4 bases in intron2 and 11 bases in exon3. Interestingly, another PNA which targets the 3' splice site of intron3 and is complementary to 4 bases in intron3 and 11 bases in exon4, exhibited both exon skipping as well as intron retention.^52^

Duchenne muscular dystrophy (DMD) is a lethal disease caused by point mutations in the dystrophin gene due to stop codon in exon 23 out of 79 exons. This early termination leads to complete loss of the functional muscle protein dystrophin throughout the body, which induces muscle complications. Although two FDA-approved drugs are in clinical use to treat DMD pateints, exon-skipping antisense oligonucleotide is still a promising approach in restoring reading frame and functional dystrophin protein. Preclinical studies revealed that PNAs are very promising method to induce splice correction by exon skipping in pre-mRNA of the DMD in mdx mice through intramuscular and systemic PNA delivery. PNA target to the intron22/exon23 junction site can induce skipping of exon 23 and produce a shortened protein still in reading frame shift with adequate activity to restore muscle function in a mouse (mdx) model. Also, it has been shown that single intramuscular administration of PNA could efficiently trigger exon-skipping, inducing dystrophin expression along with significant numbers of dystrophin-positive fibers in muscles in the mdx muscular dystrophy mouse model.^53^ In parallel, identically designed experiment, intravenously repeated injections of high dose of PNA in mdx mice, elicited exon skipping accompanied by systemic therapeutic level of dystrophin expression and functional improvement with phenotypic rescue of mdx mice without major detectable toxicity. Based on the findings, PNA-base strategy is indeed a potent and promising applicable approach for DMD exon-skipping therapeutics, achieving restoration of functional dystrophin protein.^54^

## Therapeutic approach of PNA as a gene editing agent

Available treatment for monogenic blood disorders such as sickle cell anemia and thalassemia is allogenic transplantation. However, limited accessibility of histocompatible donors along with associated side effects offers alternative strategies such as gene-targeted oligonucleotides with therapeutic potential.^55^ In support of this statement, Rogers and his colleague tested the possibility of PNA as a gene editing agent in hematopoietic progenitor cells in a mice model to stimulate site-specific mutations while retaining differentiation capability of Hematopoietic stem cell (HSC). For systemic administration, PNA was linked to transport peptide Antp to target gene in murine. Intraperitoneally injected PNAs were targeted to reporter gene (supFG1) which was chromosomally integrated in transgenic mice. Results showed mutations in the supFG1 gene and higher mutation frequencies were observed within the PNA-binding site, demonstrating that HSCs can be chromosomally changed by systemically administered PNA. It shoud be adresssed that gene modification was observed in all the hematopoietic progenitors, including erythroid, myeloid and lymphoid cell lineages. These findings clearly revealed the clinical importance of *in vivo* HSCs gene therapy with a PNA-mediated approach in a stem cell population. This approach can be a new applicable therapeutic agent providing gene therapy in systemic diseases that cannot be manipulated outside the body.^56^

As mentioned above, triplex-forming PNAs can correct the mutant gene by engaging the cell’s own DNA repair machinery via triggering recombination in the presence of a single stranded DNA template.^57^ In an analogous study, gene-targeting strategy through intravenous injection of biodegradable nanoparticle containing triplex forming PNA (PNA-DNA-PNA) plus donor DNAs were used to edit hematopoietic stem cells in HSC engrafted mice. The results demonstrated modification of the human CCR5 gene in multiple organs in the mice, which is clinically relevant to human cells. To demonstrate the versatility of this approach, they administered nanoparticles carrying triplex forming PNAs which were targeted to β- globin, a gene associated with thalassemia disease. Analysis showed single base-pair modification in the second intron of the b-globin gene in a transgenic mice.^58^ These findings are in line with *in vitro* study in which triplex forming PNAs could modify the human CCR5 gene, thus producing human immunodeficiency virus (HIV) resistant cells, as well as correcting single base-pair mutation at the intron of the *b*-globin gene, a common site of thalassemia-associated mutation.

Since PNAs can not readily cross the cell membrane and are excreted within 10–30 min after intravenous or intraperitoneal administration, hence, a delivery tool is required to access the target for *in vivo* gene editing. It has been shown that PNA and donor DNA could be efficiently encapsulated in poly (lactic-co-glycolic acid) (PLGA) nanoparticle, a FDA-approved polymer for *in vivo* drug delivery. This carrier can lead to higher efficiency in terms of gene editing both in vitro and in vivo.^59^

Bahal and McNeer reported related results by injection of nanoparticles containing next generation PNA chemistry (γPNAs) and donor DNAs targeted haematopoietic stem cells (HSCs) in a mouse model of human b-thalassemia. They observed *in vivo* gene editing of a b-thalassemia mutation in transgenic mice by simple intravenous injection of nanoparticles containing γPNA/donor DNA. In this way, the phenotype of the disease was ameliorated via elevation of blood haemoglobin levels, reduction of reticulocytosis, and reversal of splenomegaly.^60^ Taken together, these findings encourage the development of *in vivo* gene editing through PNA mediated approaches as a novel therapeutic strategy for the treatment of inherited human disorders. In a similar research, two PNAs form a bis- PNA and bind to double stranded DNA forming triplex structure by Watson-crick and hoogsteen bond. In this process, γ position of regular PNA is attached to a mini-polyethylene glycol chain to enhance solubility and binding efficacy for the DNA target. Nanoparticles containing γPNAs and 60 bp donor DNAs were administered intravenously and intra amnioticly to fetal mouse tissues in order to correct IVS2-654 mutation in the β-globin gene in a mouse model of human β-thalassemia. Obtained result revealed that this strategy can cure anemia in thalassemic mice. The result revealed that PNA-mediated gene editing is an attractive method of gene editing during pregnancy, providing higher advantage compared to after birth therapy due to accessibility to progenitor cells (HSCs), which can result in propagation of the corrected gene in all progeny cells.^61^

## Therapeutic Application of PNAs as Anti-miR agent

MicroRNAs (miRNAs) have important regulatory role in gene expression at post transcriptional stage. Additionally, upregulation of miRNAs expression is seen in several diseases remarkably in cancer, therefore they can be considered as important therapeutic targets.^62^ Notably, anti-miR PNAs can bind miR and inhibit interaction with the RISC complex through steric hindrance, thus reducing miR expression.^63^ It has been shown that PNAs have the capability to efficiently inhibit miR-155 which is expressed in the haematopoietic system in primary B cells as well as in mice model of lymphoma.^64,65^ Additionally, it has been reported that PNA can be used as an inhibitor of miR-509-3p, which is cystic fibrosis transmembrane regulating miRNA.^66^ Moreover, various MicroRNAs such as miR-210 are considerably upregulated in response to hypoxia condition, which is common in tumors development. MiR-210 may play a key role in survival and proliferation of tumor cells; it can be a good target for anti-cancer therapy. In a recently published study, γPNAs was used as a modified version of PNA to overcome limitations such as insolubility and tendency of globular formation. A regular PNA, a gamma PNA, and a control (mismatched) of 22 mer PNA were designed to target miR-210 in tumor Hela cell which grows as xenografts in immune-deficient nude mice using nanoparticle as a delivery system. Histopathological evidence showed that gamma PNAs targeted to miR-210, significantly suppressed tumor growth as well as increased the rate of apoptosis, compared to a regular PNA. Furthermore, expression of miR-210 is evaluated by RT-PCR in RNA extracted from the Hela tumor cells. There was a reduction in miR-210 expression levels compared to the control group. Under hypoxic condition, increase level of miR-210 is accompanied by suppression of ISCU protein which is downstream target of miR-210. Thus upon inhibition of miR-210, expression of ISCU is increased. Overall, PNA can inhibit microRNAs, offering novel approach in cancer therapy.^67^

## Application of PNAs as anti-infective agent

In recent years, the rate of multi-drug resistant bacteria has increased, so it is required to treat these bacteria via developing novel drugs. Since PNA can inhibit gene expression of target gene, it can be used as antibacterial compounds to treat patients suffering from multidrug resistant bacteria. It has been reported that antisense PNA can target a variety of genes in* Pseudomonas aeruginosa*, the most clinically common and is resistant to a wide range of antibiotics.^68^ Recent findings also addressed that antisense PNAs have potential to manupulate gene expression in *Plasmodium falciparum* through down regulation of an essential gene (PfSec13) which is responsible for viability in parasite. This alternative approach can be used to develop a novel and specific antimalarial drugs.^69,70^ Of note, PNAs have no antibacterial activity without sequence-based homology with target site. PNA can selectively bind to the conserved sequence in variety of bacterial genus and inhibit growth in broad-spectrum of bacteria. It has been revealed that PNA targeted to translation initiation region of specific genes including *Bacillus subtilis* (gram-positive bacteria) and* Escherichia coli, Klebsiella pneumoniae and Salmonella Typhimurium* (gram negative bacteria) in a mixed culture, selectively kills bacteria, so these findings open a novel opportunities for designing new therapeutic intervention for the treatment of pathogen bacteria.^71^

**Table 1:** Several examples of PNA-mediated gene therapy

**PNA**	**Target site**	**In vivo / in vitro**	**Disorder**	**Observed Effects**	**Ref.**
Antigene PNA	COL1A1 gene	Normal human fibroblast cell line	Fibroproliferative Disorders	Reduction of mRNA level	^72^
Antigene PNA	RAD51 gene	Human MM cell line H929 & a SCID-rab mouse model	Multiple Myeloma (MM)	Inhibition of RAD51 expression	^73^
Antigene PNA	MYCN gene	Rhabdomyosarcoma cell line	Rhabdomyosarcomas	Reduction of MYCN expression	^74^
Antisense PNA	COL7A1 gene	in vitro/ Primary adult fibroblast cultures	Dominant Dystrophic Epidermolysis Bullosa	Inhibition of the transcription of a mutant COL7A1	^75^
Antisense PNA	miR-155	Mice	Lymphomas	Inhibition of oncomiR-155	^76^
Antisense PNA	miR-221	Human breast cancer MCF-7 & MDA-MB-231 cell lines	Breast Tumors	Inhibition of onco miR-221	^77^
Antisense PNA	miR-509-3p	A549 cell lines	Cystic Fibrosis	Inhibition of miR-509-3p	^66^
Antisense PNA	Targeting LTR Direct Repeats of HBV RNA	HEPG2 cell line& an acute hepatitis B mouse model	Hepatitis B	Decline in HBV DNA	^78^
γPNA and donor DNA	Intron containing Mutation(the IVS2-654)	in vivo (β-globin/eGFP transgenic mouse)	β-thalassaemia	Induce DNA repair in mutaded gene	^79^
Antigene	Dystrophin Gene in Muscle Stem Cells	ex vivo/ in mdx mice, a mouse model of DMD	Duchenne muscular dystrophy	Permanently correct single-point mutations at the genomic level in stem cells	^80^

## Conclusion

Since the invention of PNA, several applications have been developed based on the unique properties of this oligomer; in particular, exploiting alteration of gene expression with focus on the antisense and antigene activity of PNA. Much attention has recently been given for possible clinical applications of PNAs in combination with drugs which are used in the clinic in cancer therapy based on *in vivo* experimental systems and clinical trials. However, in practice, great consideration is still needed in terms of *in vivo* administration such as immunogenicity, biodistribution, and other problems related to drug development.

## Acknowledgments

This review is a part of Ph.D thesis of Soheila Montazersaheb (Thesis No. : 102) at Faculty of Pharmacy, Tabriz University of Medical Sciences, Tabriz, Iran. The authors would like to acknowledge Prof. Peter Nielsen (University of Copenhagen, Denmark) for helpful comments on the manuscript.

## Ethical Issues

Not applicable.

## Conflict of Interest

There is no conflict of interest in this study.
